# 

**Published:** 1875-11

**Authors:** 


### Contents of November Number.

### ORIGINAL.

ART. I.—Abstract of the Introductory Address delivered at the Buffalo Medical College, Wednesday, Nov. 3d, 1875. By Prof. E. V. Stoddard, M. D., ........ 121

ART. II.—Notes on a case of Aphonia, treated by the method of Oliver. By W. W. Potter, M. D., ........ 135

ART. III.—Medical Society of the County of Albany, Semi-Monthly Meeting, Nov. 22d, 1875. Reported by James S. Bailey, M. D., ........ 136

### MEDICAL NOTES.

Notes on Ophthalmology. By Lucien Howe, M. D., ........ 144

### MISCELLANEOUS.

Diagnosis of the Lesion in Paraplegia, ........ 146

Case of Complete Occlusion of the Vagina, consequent upon an Abortion, .. 152

Neglected Branches of Medical Study, ........ 154

### EDITORIAL DEPARTMENT.

Obituary, Dr. M. E. Potter, ........ 156

BOOKS REVIEWED:

Transactions of the College of Physicians of Philadelphia, ........ 157

A Treatise on Human Physiology. By John C. Dalton, M. D ... 158

On Paralysis from Brain Disease. By H. Charlton Bastian, M. D., 158

The Mucous Membrane of the Uterus. By Geo. J. Engleman, A. M., M. D., ........ 159

Lindsay & Blakiston's Visiting List, ........ 160

Books and Pamphlets Received, ........ 160

